# Estimating the U.S. prevalence of chronic obstructive pulmonary disease using pre- and post-bronchodilator spirometry: the National Health and Nutrition Examination Survey (NHANES) 2007–2010

**DOI:** 10.1186/1465-9921-14-103

**Published:** 2013-10-09

**Authors:** Timothy Tilert, Charles Dillon, Ryne Paulose-Ram, Eva Hnizdo, Brent Doney

**Affiliations:** 1 Division of National Health and Nutrition Examination Surveys, National Center for Health Statistics, Centers for Disease Control and Prevention, 3311 Toledo Road, Hyattsville, MD 20782, USA; 2 National Institute for Occupational Safety and Health, Centers for Disease Control and Prevention, 1095 Willowdale Road, Morgantown, WV 26505, USA

**Keywords:** Spirometry, Bronchodilator, COPD, Prevalence, NHANES

## Abstract

**Background:**

During 2007–2010, the National Health and Nutrition Examination Survey (NHANES) conducted a spirometry component which obtained pre-bronchodilator pulmonary lung function data on a nationally representative sample of US adults aged 6–79 years and post-bronchodilator pulmonary lung function data for the subset of adults with airflow limitation. The goals of this study were to 1) compute prevalence estimates of chronic obstructive pulmonary disease (COPD) using pre-bronchodilator and post-bronchodilator spirometry measurements and fixed ratio and lower limit of normal (LLN) diagnostic criteria and 2) examine the potential impact of nonresponse on the estimates.

**Methods:**

This analysis was limited to those aged 40–79 years who were eligible for NHANES pre-bronchodilator spirometry (n=7,104). Examinees with likely airflow limitation were further eligible for post-bronchodilator testing (n=1,110). Persons were classified as having COPD based on FEV_1_/FVC < 70% (fixed ratio) or FEV_1_/FVC < lower limit of normal (LLN) based on person’s age, sex, height, and race/ethnicity. Those without spirometry but self-reporting both daytime supplemental oxygen therapy plus emphysema and/or current chronic bronchitis were also classified as having COPD. The final analytic samples for pre-bronchodilator and post-bronchodilator analyses were 77.1% (n=5,477) and 50.8% (n=564) of those eligible, respectively. To account for non-response, NHANES examination weights were adjusted to the eligible pre-bronchodilator and post-bronchodilator subpopulations.

**Results:**

In 2007–2010, using the fixed ratio criterion and pre-bronchodilator test results, COPD prevalence was 20.9% (SE 1.1) among US adults aged 40–79 years. Applying the same criterion to post-bronchodilator test results, prevalence was 14.0% (SE 1.0). Using the LLN criterion and pre-bronchodilator test results, the COPD prevalence was 15.4% (SE 0.8), while applying the same criterion to post-bronchodilator test results, prevalence was 10.2% (SE 0.8).

**Conclusions:**

The overall COPD prevalence among US adults aged 40–79 years varied from 10.2% to 20.9% based on whether pre- or post-bronchodilator values were used and which diagnostic criterion (fixed ratio or LLN) was applied. The overall prevalence decreased by approximately 33% when airflow limitation was based on post-bronchodilator as compared to pre-bronchodilator spirometry, regardless of which diagnostic criterion was used.

## Background

According to the Global Initiative for Chronic Obstructive Lung Disease (GOLD), COPD is a preventable and treatable disease characterized by airflow limitation that is not fully reversible [1]. Spirometry measurements can be used to define COPD, specifically the forced expiratory volume in the first second (FEV1), and the FEV1 to forced vital capacity (FVC) ratio [1-3]. Accordingly, the American Thoracic Society / European Respiratory Society (ATS/ERS) standards for the diagnosis and management of patients with COPD state that spirometry should be performed in all patients suspected of COPD and is necessary for diagnosis, assessment of disease severity, and monitoring disease progression [3].

Recent estimates based on self-reported data show that 15 million Americans aged 18 years and older have been diagnosed with COPD [4]. Estimating the prevalence of COPD can, however, be challenging and may vary based on the diagnostic method (e.g. self-report or spirometry), the criteria used for defining COPD (e.g. GOLD, ATS/ERS), and the age group analyzed (e.g., 18 years and older or 40 years and older). Estimates will also vary if pre-bronchodilator or post-bronchodilator spirometry results are used with the GOLD guidelines advocating the use of post-bronchodilator spirometry [1] while the ATS/ERS guidelines support the use of pre-bronchodilator spirometry [5]. The GOLD criteria with the fixed cutoff may be simpler to use in daily clinical practice [6,7], however, studies suggest that it overestimates disease burden in the elderly [8,9]. While the ATS/ERS guidelines may be better suited to capture age-related decline in pulmonary function, their use is more computationally intensive and requires appropriate, population-based, reference equations for the interpretation of pulmonary function tests [5].

During 2007–2010, the U.S. National Health and Nutrition Examination Survey (NHANES) conducted a spirometry component which obtained pre-bronchodilator and post-bronchodilator pulmonary lung function data on survey participants 6–79 years of age. Data on respiratory symptoms and diseases were also collected. These data offer a unique opportunity to examine multiple approaches to estimating the prevalence of COPD in a large nationally representative sample.

The main objectives of this study were 1) to estimate the prevalence of COPD in the United States based on pre-bronchodilator and post-bronchodilator spirometry measurements and according to the operational definitions in the two major published guidelines: ATS/ERS and GOLD [1,5,10] and 2) to examine the potential impact of nonresponse on the estimated prevalence.

## Methods

NHANES is a cross-sectional survey of the civilian, non-institutionalized U.S. population conducted by the National Center for Health Statistics of the Centers for Disease Control and Prevention [11]. Data were collected via household interviews and standardized physical examinations in specially equipped mobile examination centers (MEC). The NHANES survey samples are selected through a complex, multistage, probability design. Each annual sample is nationally representative, however NHANES data are publicly released for 2-year survey periods to protect confidentiality and increase statistical reliability. The 2007–10 NHANES survey cycles oversampled major U.S. demographic subgroups including Hispanic and non-Hispanic black persons, low income white persons, and persons aged 80 years and older. The procedures to select the sample and conduct the interview and examination have been specified elsewhere [12]. Informed consent was obtained from all participants and the National Center for Health Statistics Research Ethics Review Board approved the protocol.

This study was based on analysis of NHANES 2007–2008 and 2009–2010 data for participants 40–79 years. During 2007–2010, 9,985 persons aged 40–79 years were eligible for the survey, 7296 (73%) were interviewed and 7104 (71%) attended the NHANES MEC exam and were invited to participate in the spirometry component, which included pre-bronchodilator and post-bronchodilator lung function assessment. Of the 7104 eligible, 649 participants were excluded from spirometry for safety reasons: current chest pain or pain with forceful expiration, currently taking daytime supplemental oxygen, had recent surgery of the eye, chest or the abdomen; had a recent heart attack, stroke, tuberculosis exposure, hemoptysis, a history of detached retina or pneumothorax. An additional 632 participants did not receive spirometry due to limited time available in the MEC (n=337), subject refusals (n=113), or some other reason (n=182). In total, 5,823 adults aged 40–79, or 82% of those eligible, received spirometry.

**Figure 1:**
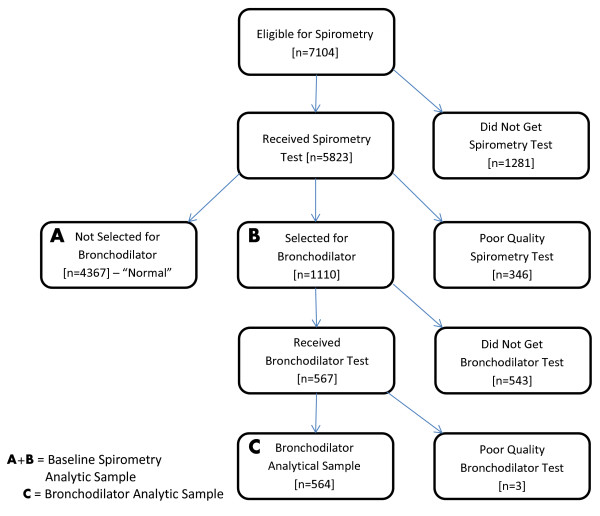
Flow diagram of the study population.

Of these 5,823 examinees, 1110 were eligible for the bronchodilator study based on pre-bronchodilator spirometry values that indicated possible airflow obstruction per ATS/ERS or GOLD criteria. Possible airflow obstruction, per the ATS/ERS criterion, was defined as a pre-bronchodilator FEV1/FVC ratio less than the lower limit of normal (LLN) representing the lower 5^th^ percentile based on person’s age, sex, height, and race/ethnicity [5]. LLN values were determined using normative reference equations developed from NHANES III data by Hankinson et al. [13]. Possible airflow obstruction, per the GOLD criterion, was a pre-bronchodilator FEV1/FVC ratio less than 70%. Of the 1110 who qualified for post-bronchodilator testing, 238 did not perform the post-bronchodilator test due to subject refusals or limited time available in the MEC. An additional 305 were excluded due to safety reasons which included active cardiovascular disease (uncontrolled blood pressure, irregular pulse on examination, taking medication for major arrhythmia, having an implanted defibrillator, or history of congenital heart disease) or taking certain prescription medications (a monoamine oxidase inhibitor, an anticonvulsant, a tricyclic antidepressant plus current treatment for cardiac disease, or potassium lowering drugs). Examinees were also excluded from bronchodilator administration if they had already recently taken a β2-adrenergic bronchodilator to avoid exceeding FDA recommended doses, or if they had had a previous adverse reaction to albuterol. Also, women who were pregnant or breastfeeding were excluded. In total, 543 adults aged 40–79 did not receive the bronchodilator test while 567, or 51% of those eligible, received the test (Figure 1).

Among those who received the pre-bronchodilator spirometry test (n=5823) and the post-bronchodilator spirometry test (n=567), 94% and 100%, respectively, had acceptable quality data for analysis. Acceptable quality was defined as either meeting or exceeding the ATS data quality standard [14] of 3 acceptable and 2 reproducible spirometric curves with 2 observed values for both FEV1 and FVC within 150 ml (87% of all tests), or a lesser standard where there were 2 curves where FEV1 values were within 200 ml, and FVC values were within 200 ml (7% of all tests). The final pre-bronchodilator and post-bronchodilator analytic samples were 5477 and 564, respectively.

### Spirometry testing

Spirometry testing was performed in accordance with recommendations of the ATS [14] using Ohio 822/827 dry-rolling seal volume spirometers with in-line biological filters (A-M Systems PFT Filter Kit B) to minimize infection risks. These were the same spirometers that were used in the NHANES III (1988–1994) spirometry testing. Spirometry was almost always performed in the standing position unless the participant had a physical limitation. Participants were coached on proper head and chin position and used a nose clip to prevent air leaks during testing. They were then instructed to perform a series of maximal forced expiratory maneuvers using standard technique. Testing for an individual continued until he/she was able to achieve a reproducible spirogram, or until a maximum of eight spirometry curves had been obtained, or until the participant could not continue. The overall goal was for the participant to achieve three acceptable exhalation maneuvers by ATS criteria in which the two highest values for the FVC and the FEV1 (each taken from an acceptable forced expiratory maneuver) showed minimal variability: i.e. the two largest FVC values taken from 2 acceptable curves should agree within 150 ml, and similarly for the two largest values for the FEV1 [15].

If a participant was selected for bronchodilator testing, the MEC physician explained the testing procedure, obtained informed consent and administered albuterol. Albuterol administration consisted of 2 puffs of 108 micrograms of albuterol sulfate each, taken one minute apart, from a PROAIR HFA™ inhalation aerosol, meter-dosed inhaler (MDI) and through an AeroChamber MAX™ spacer. Spirometric testing was then repeated. Spirometric testing procedures for the pre-bronchodilator exam and the post-bronchodilator exam were identical. For detailed descriptions of the testing protocol and quality control procedures, refer to the NHANES 2009–10 Respiratory Health Spirometry Procedure manual [15].

Using both pre- and post-bronchodilator spirometry values, participants were defined as having evidence of COPD based on either the fixed ratio (FEV1/FVC < 0.70) or LLN (FEV1/FVC < LLN) criteria.

### Other assessments of COPD

Prior to receiving the initial pre-bronchodilator spirometry exam, participants were asked if they currently had a breathing problem that required the use of supplemental oxygen during the daytime. Persons on daytime supplemental oxygen who also reported health care provider- diagnosed emphysema or current chronic bronchitis were additionally classified as having evidence of COPD.

### COPD severity

Severity was calculated for both pre- and post-bronchodilator test results. To ensure reliability of estimates (relative standard errors ≤ 30%) and to maximize comparability between diagnostic criteria, severity stages were combined into three categories. Using the fixed ratio criterion, participants were categorized as having mild (FEV1≥80% predicted), moderate (50%≤FEV1<80% predicted), or severe/very severe (FEV1<50% predicted) disease per the GOLD guidelines [1]. Using the LLN criterion, participants were categorized as having mild (FEV1>70% predicted), moderate and moderately severe (50%≤FEV1≤70% predicted), or severe/very severe (FEV1<50% predicted) disease per the ATS/ERS guidelines [5].

Percent of predicted FEV1 was defined as the observed FEV1 value divided by the predicted FEV1 value estimated for a person of the same age, gender, race/ethnicity, and height using race-specific reference equations for the U.S. population [13]. The U.S. population predicted values are available online and the method used to derive them described in the literature [13]. Reference values are available for non-Hispanic white, non-Hispanic black, and Mexican Americans. In this study, for the “other” race category, we applied a correction factor of 0.88 to the corresponding values for non-Hispanic whites, which has been previously published as an adjustment factor for Asian participants [16]. For the “other Hispanic” group, we applied the predicted values for Mexican Americans.

Participant’s height was measured using a standard protocol in the NHANES Anthropometry exam component. Data on sixteen persons with missing measured height values were imputed using a single imputation linear regression model with the following predictors: forced vital capacity, gender, ethnicity, and self-reported height.

### Other measurements

Demographic data were collected during the household interview. Age was categorized as 40–59 and 60–79 years. Self-reported race and Hispanic origin were categorized as non-Hispanic white, non-Hispanic black, Mexican American, other Hispanic, and “other”. Participants in the “other Hispanic” and “other” categories are included in overall estimates, but results for these groups are not reported separately.

### Statistical methods

Statistical analyses were performed using STATA™ version 12.1 (StataCorp, College Station, TX). Examination sample weights were used to account for differential probabilities of selection and the complex NHANES sample design and to obtain prevalence estimates and their standard errors that were representative of the non-institutionalized U.S. population ages 40–79 years. Age adjustment was carried out by the direct method using the year 2000 Census Bureau projections for the U.S. civilian, non-institutionalized population using 2 age groups: 40–59 and 60–79. Standard errors were estimated by Taylor series linearization. Chi-square tests were performed to examine the association between covariates and exam completion status. All estimates presented have a relative standard error ≤30% [17].

For the estimates based solely on pre-bronchodilator data, the examination weights of those persons defined as responders (n=5532) were adjusted, using response propensity scores, to equal the examination weight totals of those eligible for pre-bronchodilator testing (n=7104). Responders to pre-bronchodilator spirometry were defined as those having a complete, acceptable quality, pre-bronchodilator spirometry exam or reporting daily supplemental oxygen use plus self-reported, health care provider COPD diagnosis (ever had emphysema or current chronic bronchitis). Twenty-three percent of those eligible for pre-bronchodilator spirometry did not satisfy either of these requirements and were classified as non-responders to pre-bronchodilator spirometry. To compensate for the potential impact of nonresponse to pre-bronchodilator spirometry testing on the prevalence estimates, we adjusted the original examination sampling weights for responders using the inverse of the respondent's predicted response probability (propensity score) obtained from a logistic regression model as the examination weight adjustment factor [18-20]. As there were significant differences between respondents and nonrespondents in a number of covariates including age, race, and self-reported COPD diagnosis, the logistic model used for all the reweighting adjustments included the demographic and outcome-related covariates of age, race, gender, self-reported COPD diagnosis, number of years smoked, and self-reported current asthma status as the independent variables with response/nonresponse to pre-bronchodilator spirometry testing as the binary dependent outcome.

For the estimates obtained using pre- and post-bronchodilator data, we employed a 2-stage re-weighting adjustment to account for nonresponse at the level of both pre-bronchodilator and post-bronchodilator spirometry. In the first stage, the examination weights of the post-bronchodilator responders (n=564) were adjusted, using the inverse of the modeled response propensity scores, to equal the examination weight totals of those eligible for post-bronchodilator testing (n=1110). Responders for post-bronchodilator spirometry were defined as those having a complete, acceptable quality, post-bronchodilator spirometry exam while non-responders to post-bronchodilator spirometry were those without. In the second stage, this reweighted post-bronchodilator responder group (n=564) was combined with those who completed baseline spirometry and had spirometric results showing no indication of lung obstruction (n=4367) and those who reported daily supplemental oxygen use plus self-reported, health care provider COPD diagnosis (n=55). The weights for this combined group (n=4986), now representing the full complement of post-bronchodilator outcomes, were adjusted using the inverse of the modeled response propensity scores to equal the examination weight totals of all those eligible for spirometry (n=7104).

To assess the reliability of the estimates obtained using the 2-stage, pre- and post-bronchodilator reweighting, we also multiply imputed the missing post-bronchodilator data to account for post-bronchodilator nonresponse then applied the adjusted examination weights used for the estimates based solely on pre-bronchodilator data to account for pre-bronchodilator nonresponse. Post-bronchodilator FEV1 and FVC values were multiply imputed using a chained equation model with each resulting predicted FEV1 value divided by its corresponding predicted FVC value to generate a predicted post-bronchodilator FEV1/FVC ratio. The chained equation model to predict post-bronchodilator FEV1 and FVC values included the following predictors: impaired basic functional activities, had current respiratory illness, eosinophil count, mean FENO measure (exhaled nitric oxide), C-reactive protein level, number of years smoked, cotinine level, number of pack-years smoked, pre-bronchodilator FVC value, pre-bronchodilator FEV1 value, prior diagnosis of asthma, current smoker, age, race, gender, height, self-reported COPD diagnosis (emphysema or current bronchitis), chronic cough, chronic phlegm, atopy (allergic response) indicator, number of wheezing/whistling attacks in past year, low level of education (less than 9^th^ grade), and high level of education (college graduate or above). As this was a predictive model and not an explanatory model, we were not concerned with multicollinearity or its effect on the individual coefficient estimates. Rather, our goal was maximally informed predictions so, for this reason, we included a number of similar predictors in the model such as current smoker, number of years smoked, number of pack-years smoked, and cotinine level. Forty imputations of the missing post-bronchodilator data were used to produce prevalence estimates as this was the number determined to minimize preventable statistical power falloff [21].

## Results

Characteristics of persons eligible for pre- and post-bronchodilator spirometry are summarized in Table 1. The overall exam completion rate for pre-bronchodilator spirometry was 77.1 percent. 1,627 eligible individuals were excluded from the analysis due to safety reasons, not having quality spirometry results or other non-response. Spirometry completion rates were higher among 40–59 year olds (81.4%) than 60–79 year olds (72.0%); p<0.001, progressively higher with more education (Less than 12^th^ grade, 69.2%; HS grad/equivalent, 78.1%; Some college or AA degree, 80.9%; College graduate or above, 84.1%; p<0.001), and higher for those with no self-reported COPD diagnosis (yes, 55.2%; No, 78.5%; p<0.001). There were also significant differences in spirometry completion rates with respect to race/ethnicity (Non-Hispanic white, 80.4%; Non-Hispanic black, 72.8%; Mexican-American, 75.9%; p<0.001).

**Table 1:** Distribution of persons eligible for spirometry and examined

	**Pre-bronchodilator spirometry**				**Post-bronchodilator spirometry**			
	**Eligible**^**a **^**persons**		**Examined**^**b **^**persons**		**Eligible**^**c **^**persons**		**Examined**^**d **^**persons**	
	**n**	**Percent (unweighted)**	**Percent of eligible persons (unweighted)**	**Chi-square**^**e **^**p-value**	**n**	**Percent (unweighted)**	**Percent of eligible persons (unweighted)**	**Chi-square**^**e **^**p-value**
Total	7,104	100.0	77.1		1,110	100.0	50.8	
Gender				0.086				0.001
Male	3,495	49.2	78.0		675	60.8	54.7	
Female	3,609	50.8	76.3		435	39.2	44.8	
Race and ethnic origin				<.001				0.092
Non-Hispanic white	3,362	47.3	80.4		705	63.5	53.2	
Non-Hispanic black	1,437	20.2	72.8		195	17.6	42.1	
Mexican American	1,217	17.1	75.9		99	8.9	52.5	
Age				<.001				<.001
40–59	3,839	54.0	81.4		455	41.0	58.2	
60–79	3,265	46.0	72.0		655	59.0	45.6	
Education				<.001				<.001
Less than 12th grade	2,270	32.0	69.2		331	29.9	44.4	
HS Grad/GED/Equivalent	1,655	23.3	78.1		301	27.2	46.8	
Some College or AA degree	1,773	25.0	80.9		272	24.6	56.6	
College Graduate or above	1,395	19.7	84.1		204	18.4	59.8	
Smoking status				0.373				0.022
Current smoker	1,479	20.8	75.5		378	34.1	45.2	
Past smoker	2,110	29.7	77.9		414	37.3	50.7	
Never smoked	3,512	49.4	77.3		317	28.6	57.7	
Previous Diagnosis of COPD^f^				<.001				<.001
No	6,680	94.0	78.5		994	89.6	53.2	
Yes	424	6.0	55.2		116	10.5	30.2	

^a^Number of persons aged 40–79 years who attended the NHANES MEC exam, 2007-10.

^b^Percent of those eligible with a complete pre-bronchodilator spirometry examination and acceptable quality exam results by ATS/ERS Criteria or had a minimum of 2 curves where FEV1 values were within 200 ml and FVC values were within 200 ml. This combination is denoted in the data as SPXNSTAT=1.

^c^Subset of those persons with a complete, acceptable quality pre-bronchodilator spirometry examination that have results suggestive of airflow obstruction (pre-bronchodilator FEV1/FVC < .7 or < LLN)

^d^Percent of those eligible with a complete post-bronchodilator spirometry examination and acceptable quality exam results (minimum 2 curves where FEV1 values were within 200 ml and FVC values were within 200 ml). This combination is denoted in the data as SPXBSTAT=1.

^e^Chi-square values computed for differences between examined and non-examined (eligible less examined) persons.

^f^Self-reported, health care provider diagnosed COPD assessed by an affirmative response to either ever having emphysema or still having chronic bronchitis.

Of the 1,110 persons eligible for the post-bronchodilator spirometry, 564 persons had complete, acceptable quality bronchodilator results (50.8% response rate). Mirroring the baseline spirometry completion rates, post-bronchodilator completion rates were higher among 40–59 year olds (58.2%) than 60–79 years olds (45.6%); p<0.001, progressively higher with more education (Less than 12^th^ grade, 44.4%; HS grad/equivalent, 46.8%; Some college or AA degree, 56.6%; College graduate or above, 59.8%; p<0.001), and higher for those with no self-reported COPD diagnosis (yes, 30.2%; No, 53.2.5%; p<0.001). In contrast to the baseline spirometry, more men completed the post-bronchodilator exam than women (54.7% vs. 44.8%; p<0.001) and there were also significant differences in post-bronchodilator completion rates by smoking status (Current smoker, 45.2%; Past smoker, 50.7%; Never smoked, 57.7%; p<.05).

Prevalence estimates of COPD based on the fixed ratio criterion are presented in Table 2. When applying the fixed ratio criterion to pre-bronchodilator spirometry values, the overall prevalence of COPD among adults aged 40–79 years was 20.3% (SE=1.1) when using the original examination weights and 20.9% (SE=1.1) with non-response adjusted weights. The prevalence estimates for demographic subgroups were similar when using the original weights and the nonresponse adjusted weights (0.1-0.6% differences). Regardless of which survey weights were used, about 11% of adults aged 40–79 years had mild disease, 8% had moderate, and 1% had severe/very severe COPD.

**Table 2:** **COPD prevalence** (%)* **in major demographic subgroups using the fixed ratio criterion** (**FEV1**/**FVC**<**0**.**70**)

	**Fixed ratio criterion (FEV1/FVC<0.70)**				
	**Estimates based on pre**- **bronchodilator spirometry data**^**a**^		**Estimates based on pre and post**-**bronchodilator spirometry data**^**b**^		
	**Original exam weights (n = 5532)**	**Weights adjusted for non-response (n = 5532)**	**Original exam weights (n = 4986)**	**Pre-BR and Post-BR weights adjusted for non-response (n = 4986)**	**Post-BR missing data imputed then Pre-BR weights adjusted for non-response (n = 5532)**
Overall	20.3 (1.1)	20.9 (1.1)	8.3 (0.7)	14.0 (1.0)	13.7 (0.8)
Stage I (Mild)^c^	10.9 (0.7)	11.0 (0.7)	5.0 (0.6)	7.9 (0.8)	7.2 (0.5)
Stage II (Moderate)^c^	7.8 (0.6)	8.0 (0.6)	2.4 (0.2)	4.7 (0.5)	5.0 (0.4)
Stage III and IV (Severe/Very Severe)^c^	1.1 (0.2)	1.2 (0.2)	0.3 (0.1)	0.7 (0.2)	0.8 (0.2)
Age					
40–59	15.4 (1.2)	15.6 (1.2)	6.2 (0.7)	9.4 (1.0)	9.2 (0.9)
60–79	30.7 (1.3)	31.2 (1.3)	13.3 (1.1)	23.0 (1.7)	22.6 (1.2)
Gender					
Male	24.1 (1.2)	24.8 (1.3)	11.4 (0.9)	17.4 (1.2)	17.4 (1.0)
Female	16.7 (1.3)	17.3 (1.3)	5.4 (0.6)	10.8 (1.3)	10.4 (0.8)
Race and ethnic origin					
Non-Hispanic white	22.5 (1.2)	22.9 (1.2)	9.5 (0.8)	15.0 (1.1)	14.9 (0.9)
Non-Hispanic black	17.4 (1.3)	18.0 (1.4)	6.9 (0.9)	14.1 (2.0)	12.8 (1.3)
Mexican-American	10.1 (.7)	10.4 (0.7)	2.7 (0.6)	5.4 (1.1)	5.8 (0.8)

*Standard errors of the estimates are given in parentheses.

Adults Aged 40–79 Years: NHANES 2007-10.

^a^Estimates based on pre-bronchodilator included those who completed the pre-bronchodilator test plus those persons excluded from pre-BR spirometry who had a medical diagnosis of emphysema or chronic bronchitis plus used daytime supplemental oxygen therapy.

^b^Estimates based on pre and post-bronchodilator included those who completed the pre-bronchodilator test and were not selected for the post-bronchodilator test (no disease), plus those persons who completed the post-bronchodilator test, plus those persons excluded from pre-BR spirometry who had a medical diagnosis of emphysema or chronic bronchitis plus used daytime supplemental oxygen therapy. The 546 persons who did not complete the post-bronchodilator test are included in the numerator of the imputed estimates as their post-bronchodilator data was multiply imputed.

^c^Spirometric classification used the following cutoffs per GOLD specifications: Mild (Stage 1): PPFEV1 ≥ 80%, Moderate (Stage 2): 50% ≤PPFEV1 < 80%, Severe and Very severe (Stages 3 and 4): PPFEV1 < 50%.

When applying the fixed ratio criterion to post-bronchodilator spirometry values, the overall prevalence of COPD was 8.3% (SE=0.7) using the original examination weights. After adjusting for nonresponse by imputation or reweighting, the post-bronchodilator estimate using the fixed ratio was 13.7% (SE=0.8) and 14.0% (SE=1.0), respectively. Estimates were similar for demographic subgroups and disease severity regardless of which non-response adjustment approach was used, that is imputation or reweighting. Specifically, about 7.5% were mild cases, 5% were moderate, and <1% was severe/very severe COPD.

Prevalence estimates of COPD based on the LLN criterion are presented in Table 3. Based on the LLN criterion and pre-bronchodilator spirometry values, the overall prevalence of COPD was 15.0% (SE=0.8) using the original examination weights and 15.4% (SE=0.8) using the adjusted weights. Estimates were similar for demographic subgroups and disease severity when using the original or adjusted weights. About 10% had mild, a little over 3% had moderate or moderately severe, and about 1% had severe/very severe disease. Prevalence was about 14-17% among all gender, age, and race/ethnic groups except among Mexican-American adults who had a prevalence of 8.7%.

**Table 3:** **COPD prevalence** (%)* **in major demographic subgroups using the LLN criterion** (**FEV1**/**FVC**<**LLN**)

	**LLN Criterion (FEV1/FVC<LLN)**				
	**Estimates based on pre- bronchodilator spirometry data**^**a**^		**Estimates based on pre and post**-**bronchodilator spirometry data**^**b**^		
	**Original exam weights (n = 5532)**	**Weights adjusted for non-response (n = 5532)**	**Original exam weights (n = 4986)**	**Pre-BR and Post-BR weights adjusted for non-response (n = 4986)**	**Post-BR missing data imputed then Pre-BR weights adjusted for non-response (n = 5532)**
Overall	15.0 (0.8)	15.4 (0.8)	6.1 (0.5)	10.2 (0.8)	10.2 (0.6)
Mild^c^	10.2 (0.5)	10.2 (0.5)	4.4 (0.5)	7.2 (0.7)	6.5 (0.5)
Moderate/Moderately Severe^c^	3.3 (0.3)	3.4 (0.3)	0.8 (0.2)	1.7 (0.4)	2.2 (0.3)
Severe/Very Severe^c^	1.1 (0.2)	1.2 (0.2)	0.3 (0.1)	0.5 (0.1)	0.8 (0.2)
Age					
40–59	14.0 (1.1)	14.2 (1.1)	5.4 (0.6)	8.3 (1.0)	8.1 (0.8)
60–79	17.2 (0.8)	17.8 (.8)	7.7 (0.7)	13.8 (1.3)	14.4 (0.9)
Gender					
Male	16.0 (1.0)	16.5 (1.1)	8.0 (0.7)	12.2 (1.0)	12.0 (0.9)
Female	14.1 (1.0)	14.4 (1.0)	4.3 (0.6)	8.4 (1.1)	8.6 (0.7)
Race and ethnic origin					
Non-Hispanic white	16.3 (0.9)	16.7 (0.9)	6.8 (0.6)	10.7 (0.9)	11.0 (0.8)
Non-Hispanic black	14.8 (1.2)	15.3 (1.3)	5.8 (0.8)	11.7 (1.8)	10.8 (1.2)
Mexican-American	8.4 (0.7)	8.7 (.7)	1.8 (0.4)	3.6 (0.8)	4.2 (0.7)

*Standard errors of the estimates are given in parentheses.

Adults Aged 40–79 Years: NHANES 2007-10.

^a^Estimates based on pre-bronchodilator included those who completed the pre-bronchodilator test plus those persons excluded from pre-BR spirometry who had a medical diagnosis of emphysema or chronic bronchitis plus used daytime supplemental oxygen therapy.

^b^Estimates based on pre and post-bronchodilator included those who completed the pre-bronchodilator test and were not selected for the post-bronchodilator test (no disease), plus those persons who completed the post-bronchodilator test, plus those persons excluded from pre-BR spirometry who had a medical diagnosis of emphysema or chronic bronchitis plus used daytime supplemental oxygen therapy. The 546 persons who did not complete the post-bronchodilator test are included in the numerator of the imputed estimates as their post-bronchodilator data was multiply imputed.

^c^Spirometric classification used the following cutoffs per ATS/ERS specifications: Mild: PPFEV1 > 70%, Moderate/Moderately severe: 50% ≤PPFEV1 <= 70%, Severe and Very Severe: PPFEV1 < 50%.

Applying the LLN criterion to post-bronchodilator spirometry, the overall prevalence estimates for COPD using the original examination weights was 6.1% (SE=0.5). By comparison, after multiple imputation or after reweighting, the post-bronchodilator estimates using the LLN ratio were 10.2% (SE=0.7) and 10.2% (SE=0.8), respectively. Estimates were similar for demographic subgroups and by disease severity regardless of which non-response approach was used, that is either multiple imputation or reweighting adjustment. Specifically, about 6.5 to 7% had mild disease, about 2% had moderate or moderately severe, and about 0.5% had severe/very severe disease.

## Discussion

Spirometry can be used to define COPD yet currently established guidelines (e.g., ATS/ERS and GOLD) differ in their recommendation for using spirometric measurements to do so. Specifically, the GOLD guidelines recommend using post-bronchodilator FEV1/FVC <0.70 and ATS/ERS recommends pre-bronchodilator FEV1/FVC < LLN. Applying both of these criteria to the 2007–2010 NHANES spirometry data, we found the prevalence of COPD in the U.S. for individuals aged 40 to 79 years to be 14.0% using the fixed ratio criterion with post-bronchodilator spirometry (GOLD) and 15.4% using the LLN criterion with pre-bronchodilator spirometry (ATS/ERS). Currently, no other published post-bronchodilator COPD prevalence estimates exist for the overall U.S. population. A similar study, conducted outside the U.S., which utilized fixed ratio, post-bronchodilator spirometry data in an aged 40+ population was the PLATINO study which found a 14.3% pooled prevalence across five Latin American cities [22]. This is comparable to the estimated U.S. prevalence of 14.0% we found using fixed ratio, post-bronchodilator spirometry data in a population ages 40 to 79 years.

Examination of the differences between using pre-bronchodilator and post-bronchodilator data revealed a reduction in COPD prevalence after bronchodilation, relative to before bronchodilation, of 33% using the fixed ratio criterion (pre-bronchodilator 21% vs. post-bronchodilator 14%) and 34% for the LLN criterion (pre-bronchodilator 15% vs. post-bronchodilator 10%). This puts our pre- to post-bronchodilator reduction estimates at the high end, but within, the 25-35% range of reductions in prevalence found in a number of other studies [23-26]. This finding further strengthens the growing body of evidence that COPD prevalence rates computed using post-bronchodilator spirometry are likely to be 25-35% lower than those computed using pre-bronchodilator spirometry.

Comparing results obtained based on currently established guidelines, the overall COPD prevalence rates using the GOLD post-bronchodilator and ATS/ERS pre-bronchodilator criteria were somewhat similar at 14.0% (SE=1.0) and 15.4% (SE=0.8), respectively. COPD prevalence according to disease severity stage was somewhat similar, as well (overlapping confidence intervals), with some of the residual point estimate differences explained, in part, by the different cut-points used by the GOLD and ATS/ERS systems to define COPD severity stages. For example, the GOLD criteria define mild disease as greater than or equal to a percent of predicted FEV1 (PPFEV1) of ≥0.8, whereas ATS/ERS defines mild COPD with a slightly larger range of PPFEV1 ≥0.7. As a result, the calculated ATS/ERS prevalence estimate for mild COPD (pre-bronchodilator) was 10.2% (SE=0.5), compared to the 7.9% (SE=0.8) rate for the GOLD criteria (post-bronchodilator). Conversely, for moderate disease, the GOLD moderate stage has a slightly wider range of values (.5 ≤ PPFEV1 < .8) than the ATS/ERS moderate and moderately severe stage specifications (.5 ≤ PPFEV1 ≤ .7), hence the prevalence of moderate COPD severity was expectedly higher for the (post-bronchodilator) GOLD criteria (4.7%, SE=0.5) as compared to (pre-bronchodilator) ATS/ERS criteria (3.4%, SE=0.3). When the severity is equivalent for both diagnostic criteria, as is the case with severe and very severe disease (PPFEV1 < .5), the prevalence estimates were again similar with GOLD producing an estimate of 0.7% (SE=0.2) and ATS/ERS producing 1.2% (SE=0.2).

Using the fixed ratio criterion with post-bronchodilator spirometry led to greater variability than when using the LLN criterion with pre-bronchodilator spirometry when estimating the COPD prevalence by demographic subgroups. Most notably, COPD prevalence was 14.2% among those aged 40–59 years and 17.8% among those aged 60–79 years using LLN and pre-bronchodilator data compared to the 9.4% prevalence for 40–59 year olds and 23% prevalence for 60–79 year olds using the fixed ratio and post-bronchodilator data. This appears to be a common finding, as other studies have shown that using the fixed ratio to define airflow limitation will result in more frequent diagnosis of COPD in the elderly [8,9], and less frequent diagnosis among those younger than 45 years [27].

On the other hand, some criticisms of the LLN include the lack of longitudinal studies validating the use of the LLN and the argument that its use may underestimate the true burden of disease.

In this study, disease classification and subsequent prevalence estimates are based solely on spirometric assessments with the exception of self-reported supplemental oxygen use. Using spirometry alone to classify disease is consistent with the methods used in other population based studies, however, this represents a simplified and measurable case definition in a research study setting, as opposed to an actual disease diagnosis in a clinical setting [28] and could potentially lead to disease misdiagnosis. To reduce potential misdiagnosis, the most recent GOLD committee recommendations have included evaluating symptoms and risk factors, in addition to evaluating spirometric results, when clinically diagnosing COPD [1]. The fielding of our study preceded these recommendations and, as a result, it was not possible to evaluate all the symptoms and risk factors from our survey content. As daytime supplemental oxygen use together with self-reported, health care provider-diagnosed emphysema or current chronic bronchitis is thought to be indicative of the disease with a high degree of certainty, even in the absence of a spirometric assessment, persons with these characteristics were also classified as having COPD. Since they were excluded from the exam due to supplemental oxygen use, and subsequently had no spirometric data, the disease stage for these persons could not be determined. However, given their supplemental oxygen use, it is likely they are in one of the more severe stages of disease. These supplemental classification criteria, not based on spirometry measurements, applied to a small number of cases (55) and increased the overall, strictly spirometry-based prevalence rates only by a small amount: about one half of one percentage point.

A limitation of our study was that primarily due to safety exclusions and time available for exams, we had nearly 50% nonresponse for post-bronchodilator testing. This resulted in a significant portion of potentially positive disease cases being missing due to nonresponse. To account for this missing data, estimates were produced, and compared, using two different approaches: multiple imputation of the missing data and re-weighting of those cases with valid spirometry data. Although we applied different strategies to account for non-response, the presented estimates may still be an underestimation as some of the eligible participants were excluded from the bronchodilator portion of the exam due to the recent use of a prescribed bronchodilator. Recent bronchodilator use was not accounted for in the nonresponse adjustment.

Beyond missing data, there were also other limitations in the study. Persons with no race designation on the public use file (other) were assigned predicted pulmonary values which were 88% of the corresponding predicted pulmonary values for whites. This adjustment factor was originally derived for Asians and, since some participants in this “other” group may be other than Asian, this correction factor may cause some misclassification of COPD severity stage. Similarly, application of predicted values derived from Hispanics of Mexican origin to Hispanics of non-Mexican origin could possibly cause some misclassification of COPD severity stage due to differences in smoking habits, culture, and ancestry. The more severe stages of COPD may have been further underreported by limiting our analyses to the use of acceptable quality curves (grades of A, B, or C). Curve quality was determined using both within-maneuver evaluations (no cough during first second, no extra breaths, etc.) and between-maneuver evaluations (number of acceptable spirograms, ATS-defined reproducibility criteria) [14]. As the likelihood of producing reproducible spirometric measurements may decrease with increasing severity of lung disease, the exclusion of nonreproducible tests could potentially and selectively exclude a higher proportion of persons with obstructive airways disease [29,30]. We expected that this exclusion due to lack of test reproducibility would have a negligible impact on our results. The number of persons in the poor quality spirometry curve group was relatively small (349 persons out of 7104 eligible, or 5%) and the 6.3% rate of self-reported, health care provider-diagnosed COPD (affirmative response to either having emphysema or current bronchitis) for these 349 persons was only marginally higher than the 5.2% rate found for the entire group eligible for spirometry (n=7104).

Finally, the most recent GOLD guidelines recommend the use of either the Modified British Medical Research Council (mMRC) questionnaire or the COPD Assessment Test (CAT) for assessing symptoms in patients with COPD [1]. Although NHANES does collect some respiratory symptom data, it was not a close match to the GOLD specifications. ATS guidance regarding COPD symptom criteria is even less specific than the GOLD criteria. We therefore limited the scope of our study primarily to spirometric definitions of COPD to make valid comparisons between ATS and GOLD criteria. It should be noted, however, that the multiple imputation models to predict FEV1 and FVC values included available symptom information to generate maximally informed prediction equations.

In conclusion, the overall prevalence of COPD among U.S. adults aged 40–79 years ranged from 10.2% to 20.9% based on whether pre- or post-bronchodilator values were used and which diagnostic criterion (fixed ratio or LLN) was applied. These estimates provide the first U.S. national estimates of impaired lung function evidence of COPD using both pre- and post-bronchodilator spirometry measurements. Using the GOLD recommended combination of a fixed ratio criterion and post-bronchodilator spirometry resulted in an overall COPD prevalence estimate of approximately 14% while using the ATS/ERS recommended combination of LLN criterion and pre-bronchodilator spirometry resulted in an overall prevalence of about 15%. The overall prevalence decreased by approximately 33% when airflow limitation was based on post-bronchodilator testing as compared to pre-bronchodilator spirometry, regardless of which diagnostic criterion was used. Due to the large percentage of missing post-bronchodilator data, the NHANES post-bronchodilator data required adjustment for nonresponse but either method of adjustment, multiple imputation or reweighting, appeared to provide similar results. Nonresponse adjustment of the pre-bronchodilator data did not lead to significant differences in computed prevalence estimates.

## Conclusions

The overall prevalence of COPD among US adults aged 40–79 years varied from 10.2% to 20.9% based on whether pre- or post-bronchodilator values were used and which diagnostic criterion (fixed ratio or LLN) was applied. The overall prevalence decreased by approximately 33% when airflow limitation was based on post-bronchodilator as compared to pre-bronchodilator spirometry, regardless of which diagnostic criterion was used.

## Competing interests

The authors declare that they have no competing interests.

## Authors’ contributions

TT conceived of the analyses, analyzed and interpreted the data, and drafted the manuscript. CD participated in the design of the study, performed quality control on both exams and data, and helped to draft the manuscript. RP provided significant conceptual input, contributed intellectual content, and helped to draft the manuscript. EH provided clinical diagnostic expertise and provided critical review of the manuscript. BD contributed significant intellectual content and provided critical review of the manuscript. All authors read and approved the final manuscript.

## References

[B1] Global Initiative for Chronic Obstructive Lung Disease (GOLD) Global Strategy for the Diagnosis, Management and Prevention of COPD 2013 http://www.goldcopd.org

[B2] Barnes TA Fromer L Spirometry use: detection of chronic obstructive pulmonary disease in the primary care setting Clin Interv Aging 2011 6 47 52 21472091 10.2147/CIA.S15164 PMC3066252

[B3] American Thoracic Society - European Respiratory Society Standards for the Diagnosis and Management of Patients with COPDhttp://www.thoracic.org/clinical/copd-guidelines/resources/copddoc.pdf

[B4] CDC Chronic obstructive pulmonary disease among adults - United States, 2011 MMWR MMWR 2012 61 938 943 23169314

[B5] Pellegrino R Viegi G Brusasco V Crapo RO Burgos F Casaburi R Coates A van der Grinten CP Gustafsson P Hankinson J Jensen R Johnson DC MacIntyre N McKay R Miller MR Navajas D Pedersen OF Wanger J Interpretative strategies for lung function tests Eur Respir J 2005 26 5 948 968 10.1183/09031936.05.00035205 16264058

[B6] Steinacher R Parissis JT Strohmer B Eichinger J Rottlaender D Hoppe UC ltenberger J Comparison between ATS/ERS age-and gender-adjusted criteria and GOLD criteria for the detection of irreversible airway obstruction in chronic heart failure Clin Res Cardiol 2012 101 8 637 645 10.1007/s00392-012-0438-0 22395777

[B7] Calverley PMA The GOLD Classification Has Advanced Understanding of COPD Am J Respir Crit Care Med 2004 170 3 211 212 10.1164/rccm.2405008 15280171

[B8] Hardie JA Buist AS Vollmer WM Ellingsen I Bakke PS Morkve O Risk of over-diagnosis of COPD in asymptomatic elderly never-smokers Eur Respir J 2002 20 5 1117 1122 10.1183/09031936.02.00023202 12449163

[B9] Celli BR Halbert RJ Isonaka S Schau B Population impact of different definitions of airway obstruction Eur Respir J 2003 22 2 268 273 10.1183/09031936.03.00075102 12952259

[B10] Bakke PS Ronmark E Eagan TM Pistelli F Annesi-Maesano I Maly M Meren M Zielinski J Viegi G Lundback B Recommendations for epidemiological studies on COPD Eur Respir J 2012 39 5 1278 1279 10.1183/09031936.00011712 22130763

[B11] National Center for Health Statistics National Health and Nutrition Examination Surveyhttp://www.cdc.gov/nchs/about/major/nhanes/datalink.htm

[B12] Zipf G Chiappa M Porter K Ostchega Y Lewis B Dostal J The National Health and Nutrition Examination Survey: Plan and Operations, 1999–2010 Vital Health Stat 2013 1 56 10 22 25078429

[B13] Hankinson JL Odencrantz JR Fedan KB Spirometric reference values from a sample of the general U.S. population Am J Respir Crit Care Med 1999 159 1 179 87 10.1164/ajrccm.159.1.9712108 9872837

[B14] Miller MR Hankinson J Brusasco V Burgos F Casaburi R Coates A Crapo R Enright P van der Grinten CP Gustafsson P Jensen R Johnson DC MacIntyre N McKay R Navajas D Pedersen OF Pellegrino R Viegi G Wanger J Standardisation of spirometry Eur Respir J 2005 26 2 319 338 10.1183/09031936.05.00034805 16055882

[B15] NHANES Respiratory Health - Bronchodilator Procedures Manual http://www.cdc.gov/nchs/data/nhanes/nhanes_07_08/Bronchodilator.pdf

[B16] Hankinson JL Kawut SM Shahar E Smith LJ Stukovsky KH Barr RG Performance of American Thoracic Society-Recommended Spirometry Reference Values in a Multiethnic Sample of AdultsThe Multi-Ethnic Study of Atherosclerosis (MESA) Lung Study CHEST 2010 137 1 138 145 10.1378/chest.09-0919 19741060 PMC2803123

[B17] National Center for Health Statistics NHANES Analytic and Reporting Guidelineshttp://www.cdc.gov/nchs/data/nhanes/nhanes_03_04/nhanes_analytic_guidelines_dec_2005.pdf

[B18] Wun LM Ezzati-Rice TM Diaz-Tena N Greenblatt J On modelling response propensity for dwelling unit (DU) level non-response adjustment in the Medical Expenditure Panel Survey (MEPS) Stat Med 2007 26 8 1875 84 10.1002/sim.2809 17206601

[B19] Kalton G Flores-Cervantes I Weighting methods Journal of Official Statistics-Stockholm 2003 19 2 81 98

[B20] Potter F Grau E Williams S Diaz-Tena N Carlson BL An application of propensity modeling: Comparing unweighted and weighted logistic regression models for nonresponse adjustments. Proceedings of the Survey Research Methods Section. American Statistical Association http://www.amstat.org/sections/srms/Proceedings/y2006f.html

[B21] Graham JW Olchowski AE Gilreath TD How many imputations are really needed? - Some practical clarifications of multiple imputation theory Prev Sci 2007 8 3 206 213 10.1007/s11121-007-0070-9 17549635

[B22] Menezes AM Pérez-Padilla R Hallal PC Jardim JR Muiño A López MV Valdivia G Pertuze J De Oca MM Tálamo C Worldwide burden of COPD in high- and low-income countries. Part II. Burden of chronic obstructive lung disease in Latin America: the PLATINO study Int J Tuberc Lung Dis 2008 12 7 709 12 18544192

[B23] Johannessen A Omenaas ER Bakke PS Gulsvik A Implications of reversibility testing on prevalence and risk factors for chronic obstructive pulmonary disease: a community study Thorax 2005 60 10 842 7 10.1136/thx.2005.043943 16085729 PMC1747202

[B24] Pérez-Padilla R Hallal PC Vázquez-García JC Muiño A Máquez M López MV De Oca MM Tálamo C Valdivia G Pertuzé J Jardim J Menezes AM Impact of bronchodilator use on the prevalence of COPD in population-based samples COPD 2007 4 2 113 20 10.1080/15412550701341012 17530504

[B25] Shirtcliffe P Weatherall M Marsh S Travers J Hansell A McNaughton A Aldington S Muellerova H Beasley R COPD prevalence in a random population survey: a matter of definition Eur Respir J 2007 30 2 232 9 10.1183/09031936.00157906 17666557 PMC2516341

[B26] Vollmer WM Enrigh PL Buist AS Gislason P Burney P Gulsvik A Kocabas A Comparison of spirometry criteria for the diagnosis of COPD: results from the BOLD study Eur Respir J 2009 34 3 588 597 10.1183/09031936.00164608 19460786 PMC3334278

[B27] Cerveri I Corsico AG Accordini S Niniano R Ansaldo E Anto JM Kunzli N Janson C Sunyer J Jarvis D Svanes C Gislason T Heinrich J Schouten JP Wjst M Burney P De Marco R Underestimation of airflow obstruction among young adults using FEV(1)/FVC < 70% as a fixed cut-off: a longitudinal evaluation of clinical and functional outcomes Thorax 2008 63 12 1040 1045 10.1136/thx.2008.095554 18492741

[B28] Menezes AM Pérez-Padilla R Jardim JB Muiño A López MV Valdivia G De Oca MM Tálamo C Hallal PC Victora CG Chronic obstructive pulmonary disease in five Latin American cities (the PLATINO study): a prevalence study LANCET 2005 366 9500 1875 1881 10.1016/S0140-6736(05)67632-5 16310554

[B29] Mannino DM Buist AS Petty TL Enright PL Redd SC Lung function and mortality in the United States: data from the First National Health and Nutrition Examination Survey follow up study Thorax 2003 58 388 393 10.1136/thorax.58.5.388 12728157 PMC1746680

[B30] Halbert RJ Natoli JL Gano A Badamgarav E Buist AS Mannino DM Global burden of COPD: systematic review and meta-analysis Eur Respir J 2006 28 3 523 32 10.1183/09031936.06.00124605 16611654

